# CRISPR Gene-Editing Models Geared Toward Therapy for Hereditary and Developmental Neurological Disorders

**DOI:** 10.3389/fped.2021.592571

**Published:** 2021-03-11

**Authors:** Poh Kuan Wong, Fook Choe Cheah, Saiful Effendi Syafruddin, M. Aiman Mohtar, Norazrina Azmi, Pei Yuen Ng, Eng Wee Chua

**Affiliations:** ^1^Drug and Herbal Research Centre, Faculty of Pharmacy, Universiti Kebangsaan Malaysia, Kuala Lumpur, Malaysia; ^2^Department of Paediatrics, Universiti Kebangsaan Malaysia Medical Centre, Kuala Lumpur, Malaysia; ^3^UKM Medical Molecular Biology Institute, Universiti Kebangsaan Malaysia, Kuala Lumpur, Malaysia

**Keywords:** Clustered Regularly Interspaced Short Palindromic Repeat (CRISPR), gene-editing, gene therapy, hereditary neurological disorders, neonates, pharmacogenomics, caffeine, drug responsiveness

## Abstract

Hereditary or developmental neurological disorders (HNDs or DNDs) affect the quality of life and contribute to the high mortality rates among neonates. Most HNDs are incurable, and the search for new and effective treatments is hampered by challenges peculiar to the human brain, which is guarded by the near-impervious blood-brain barrier. Clustered Regularly Interspaced Short Palindromic Repeat (CRISPR), a gene-editing tool repurposed from bacterial defense systems against viruses, has been touted by some as a panacea for genetic diseases. CRISPR has expedited the research into HNDs, enabling the generation of *in vitro* and *in vivo* models to simulate the changes in human physiology caused by genetic variation. In this review, we describe the basic principles and workings of CRISPR and the modifications that have been made to broaden its applications. Then, we review important CRISPR-based studies that have opened new doors to the treatment of HNDs such as fragile X syndrome and Down syndrome. We also discuss how CRISPR can be used to generate research models to examine the effects of genetic variation and caffeine therapy on the developing brain. Several drawbacks of CRISPR may preclude its use at the clinics, particularly the vulnerability of neuronal cells to the adverse effect of gene editing, and the inefficiency of CRISPR delivery into the brain. In concluding the review, we offer some suggestions for enhancing the gene-editing efficacy of CRISPR and how it may be morphed into safe and effective therapy for HNDs and other brain disorders.

## Introduction

The human brain is the most complex organ in our body, consisting of a multitude of neurons communicating with each other. It is the command center that governs our bodily functions, including senses, movements, emotions, language, communication, thoughts, and memory. The intricate neural circuits of the brain are built *in utero* and continue to grow till adulthood. The process is orchestrated by a collection of genes that encode signals for triggering neural cell differentiation and migration; but many of the genes are still unknown (1, 2). Defects in these genes impair prenatal brain development and cause hereditary neurological disorders (HNDs) (3). Currently, there is no cure for most of the HNDs, as the underlying pathogenesis is often obscure and poorly understood; and effective treatments of HNDs are impeded by the blood-brain barrier (BBB) that prevents drugs from being delivered to their target sites. For many of the known HNDs, symptomatic treatments are the only feasible avenues to clinical care (4, 5). However, a major caveat is that some HNDs may not become manifest until after the neonatal period, and critical treatment opportunities may be missed (6).

Gene-editing systems, such as Zinc Finger Nucleases (ZFN) and Transcription Activator-Like Effector Nuclease (TALEN), are potentially powerful approaches for the disease-modifying treatment of HNDs (7). However, they are complex, time-consuming, and have low gene-editing efficiency. To date, Clustered Regularly Interspaced Short Palindromic Repeat (CRISPR) is the most efficient and simplest genome editing system and has been widely used in different cell types and organisms to edit single or multiple target genes (8). CRISPR can be directed to different genetic loci simply by redesigning the sgRNAs (Table 1), unlike ZFN and TALEN which would require time-consuming synthesis of new guiding proteins (9). The ease of reconstructing sgRNAs enables CRISPR to target multiple loci simultaneously, needing only an assortment of gene-specific sgRNAs. Moreover, wild-type Cas9 can be reprogrammed into catalytically inactive Cas9 (dead Cas9 or dCas9) that can modulate target gene expression when it is fused to transcriptional modifiers (10).

**Table 1:** Gene-editing glossary (62, 63).

**Term**	**Definition**
Autosomal dominant	A pattern of inheritance in which an affected individual has a copy of a mutant gene and a normal gene on a pair of autosomal chromosomes
Autosomal recessive	A pattern of inheritance in which an affected individual has a mutant gene on each autosomal chromosome
Cas9 nickase	Cas9 mutant with a single functional endonuclease domain and is only able to introduce single-stranded DNA nicks
CRISPR-associated protein 9 (Cas9)	An enzyme that cuts DNA at specific sites, guided by gRNA
Double-strand break (DSB)	A break in the DNA double helix that is formed when both strands are cut by Cas9. This is different from a single-strand break or “nick.”
Guide RNA (gRNA)	A short segment of RNA, usually 20 nucleotides, used to direct a DNA-cutting enzyme, such as Cas9, to the target location in the genome. It contains sequences which are complementary to the target sequence. It is also frequently referred to as single guide RNA (sgRNA).
Homology-directed repair (HDR)	A DNA repair mechanism that uses a template that is homologous to the site of DNA double-strand break to repair the break
Insertion/deletion (Indels)	Mutations that could disrupt an entire protein-coding frame of amino acids and abrogate gene function
Non-homologous end-joining (NHEJ)	A natural repair process used to join the two ends of a broken DNA strand. This is prone to errors where short indels are introduced.
Off-target effect	An undesired effect that occurs when Cas9 cuts at an unintended site, which typically resembles the target site
Protospacer adjacent motif (PAM)	A short segment of a few nucleotides adjacent to the sequence that is cleaved by Cas9
Ribonuclear protein complex (RNP)	A complex of gRNA and Cas9 that cuts DNA at specific sites

The number of clinical trials looking into CRISPR-based therapy, especially that of cancer, is growing, (11). Recently, a patient with Leber congenital amaurosis, a hereditary disorder that causes blindness, became the first patient to undergo *in vivo* gene editing using CRISPR (ClinicalTrials.gov identifier: NCT03872479). Although CRISPR has not yet reached the clinical stages of testing in humans with HNDs, pre-clinical results have demonstrated the efficacy of CRISPR in correcting faulty genes associated with HNDs (12). Therefore, CRISPR-based treatments could help to reduce the mortality and morbidity in neonates who suffer HNDs. A viable treatment strategy would be pre-emptive CRISPR gene editing that could prevent the causal genetic defects from developing into full-blown HNDs (6).

Besides, CRISPR can be utilized to generate models to study the effects of genetic variation on drug response (13, 14). Caffeine has a beneficial effect on the developing brain, improving cognitive outcomes in infants treated with it (15). However, caffeine sensitivity varies between neonates, possibly owing to genetic variation. The outcome of caffeine therapy was shown to be adversely affected by rs16851030, a DNA variant located in the 3'-untranslated region of the *ADORA1* gene—the target of caffeine. Individuals who are homozygous for the rs16851030 C-allele may respond better to caffeine than those who harbor the T-allele (16).

In this review, we discuss how CRISPR may progress from laboratory benches into clinics to improve neonatal care. We cover a particularly interesting but challenging subject—the management of HNDs, which affect the vulnerable, growing brains of newborns. We include only relevant HNDs that are manifest during the neonatal period and those that occur later. We also discuss how CRISPR may help us to understand the genetic basis of the variable caffeine sensitivity among neonates with apnea of prematurity, given the large amount of evidence pointing to a beneficial effect of caffeine on brain development.

## CRISPR Gene-Editing vs. Traditional Gene Therapy

**Figure 1:**
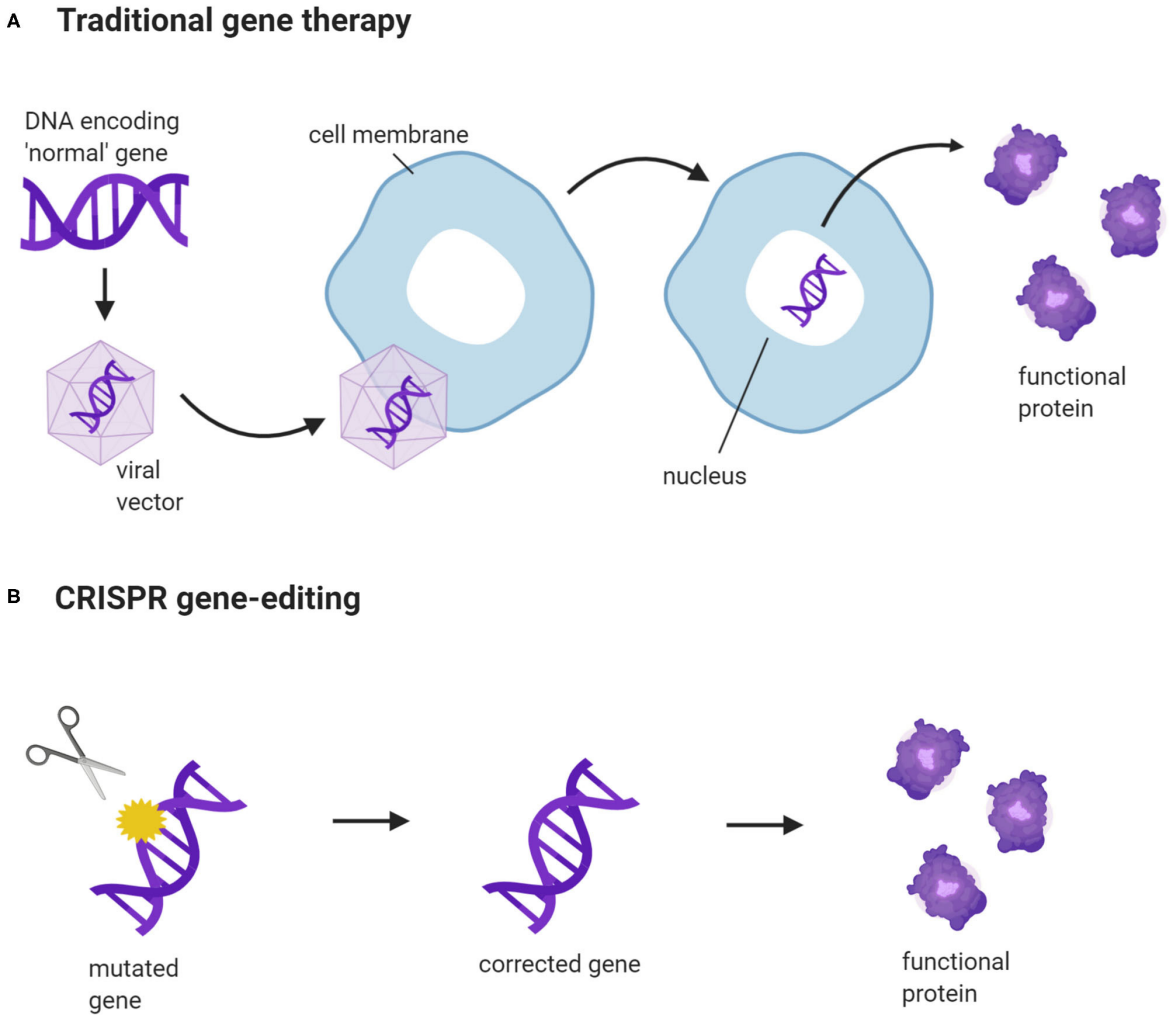
CRISPR gene-editing vs. traditional gene therapy. **(A)** A new gene construct is packaged into a viral vector. The vector binds to the cell membrane and releases the gene construct into the cell nucleus. This enables the cells to produce normal, functional proteins, encoded by the construct. **(B)** CRISPR targets a specific gene and corrects a disease-causing DNA variant. The corrected gene can now be transcribed and translated into a functional protein. (Created with BioRender.com).

Gene therapy has the potential to treat a wide range of inherited diseases, such as cystic fibrosis and muscular dystrophy (17, 18). Traditional gene therapy replaces faulty genes with the correct versions or, with the help of a vector, introduces new genes into cells to produce functional proteins (19) (Figure 1). However, not all gene constructs can fit into a vector. The gene expression system has a size limit, and large genes are difficult to package and deliver into cells (20). Traditional gene therapy works well for autosomal recessive disorders as the mechanism is straightforward. Most autosomal recessive disorders are caused by loss-of-function exonic variants, and inserting normal copies of the target gene into cells is sufficient to restore protein function (21). In contrast, autosomal dominant disorders are caused by gain-of-function exonic variants and require more elaborate gene editing. This includes an exogenous supply of functional gene constructs to restore protein function, and the use of antisense oligonucleotides and small interfering RNAs to silence the transcription of disease-causing genes (22, 23). The additional requirement for gene silencing means that repeat doses would be needed to maintain therapeutic efficacy (24). This would not be necessary with gene edits created by CRISPR, as the outcome is long-lasting.

Deaths reported following gene therapy have cast serious concerns on its safety. For example, two patients suffered liver dysfunction and died after they received high doses of adeno-associated viruses (AAV) that delivered a gene therapy for X-linked myotubular myopathy (25). An 18-year-old patient passed away 4 days after he was given an intra-artery dose of a gene therapy that was designed to remedy ornithine transcarbamylase deficiency. The cause of death was determined to be a severe immune reaction to the AAV vector that carried the corrective gene (26).

## The Mechanics of CRISPR

CRISPR is an adaptive immune defense used by archaea and bacteria against viruses (27). Upon infection by a virus, the host will integrate fragments of the viral genetic material into its genome, which will serve as memory for recognizing and destroying the virus in subsequent infections. The virus will be targeted and destroyed by the CRISPR-associated protein (Cas), an endonuclease that cleaves DNA strands. Between 2011 and 2013, substantial efforts by many researchers led to the successful repurposing of this CRISPR-Cas system to enable gene editing in eukaryotes. The mechanics of CRISPR-Cas are simple and readily reproducible outside the microbes (28, 29).

**Figure 2:**
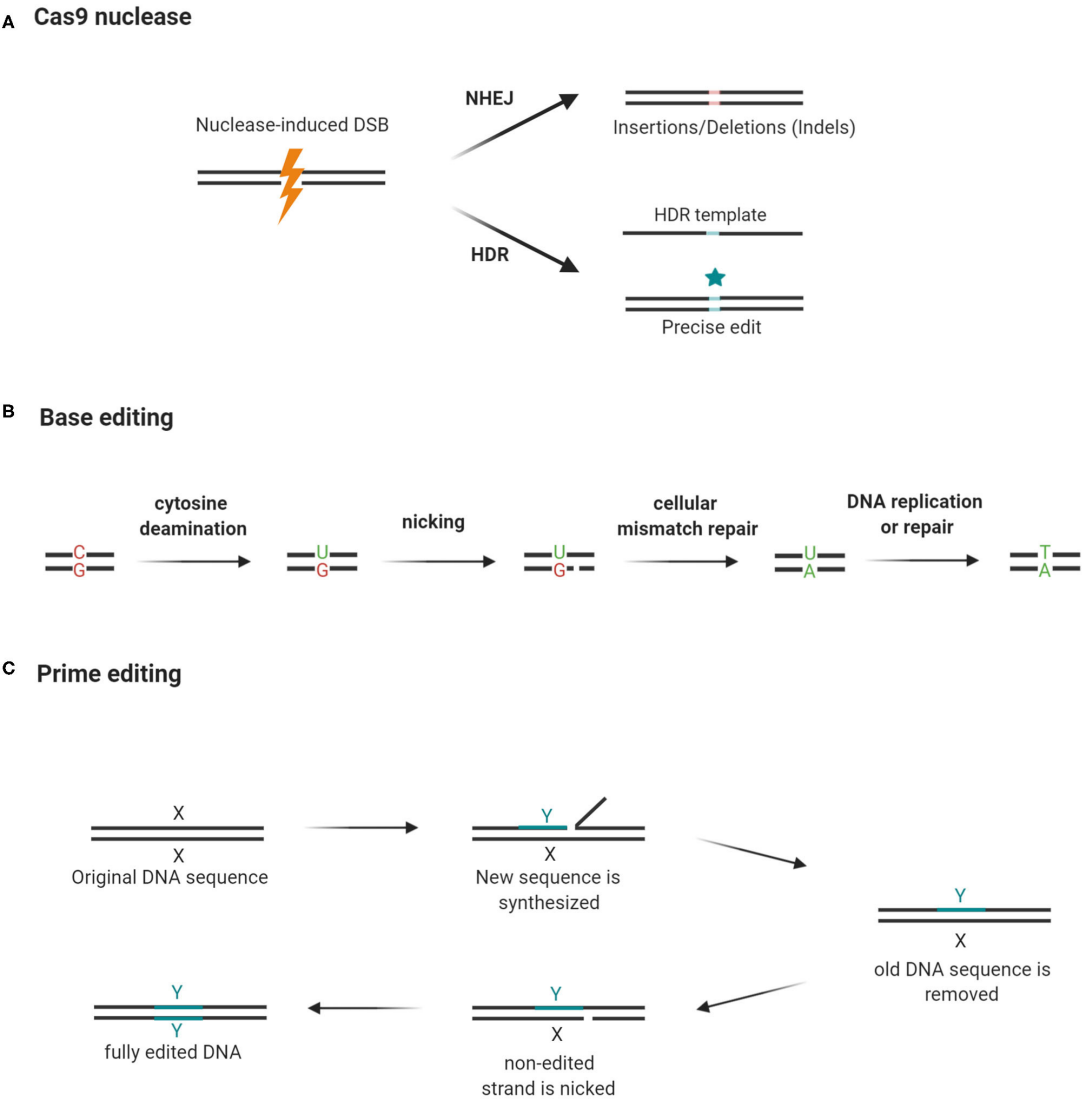
Cas9 nuclease, base editing, and prime editing. **(A)** Guide RNA guides Cas9 nuclease to cut a DNA segment that is 3 bases upstream of the PAM. The resultant double-strand break (DSB) triggers NHEJ, which may cause frame-shifting indels and abolish gene expression. By using a repair template, the repair machinery can be shifted to HDR and introduce precise edits while rejoining the broken DNA strands. **(B)** Cytidine deaminase converts cytosine to uracil while deoxyadenosine deaminase (not shown in the figure) converts adenosine to guanine. Cas9 nickase cuts the opposite strand and triggers a mismatch repair mechanism. As a result, in repairing the nick previously created by the Cas9 nickase, the cell uses the edited DNA strand as a template and copies the “mutation” into the complementary strand. **(C)** Prime editing requires a prime editor and a prime editing guide RNA (pegRNA) to modify gene sequences. The prime editor is a chimera of a Cas9 nickase and a reverse transcriptase (RT). The pegRNA guides the prime editor to the target site where editing should occur. It also carries a primer-binding site (PBS) and a short stretch of a template sequence containing the desired edit. The reverse transcriptase converts the template sequence into complementary DNA, which is then incorporated into the target site after the original DNA sequence is excised by an endogenous endonuclease. Then, the edited strand serves as a template for the repair of the unedited strand after it is nicked by Cas9 nickase. Hence, both DNA strands have the desired edit. X: original DNA sequence; Y: edited DNA sequence (Created with BioRender.com).

CRISPR has been widely used to edit single or multiple target genes in a variety of cells and organisms (8). For a detailed discussion of the mechanisms of CRISPR and the requirements for successful gene edits, the readers are referred to another review (30). A functional CRISPR toolkit needs only: (I) the Cas nuclease, commonly the Cas9, and (II) a guide RNA complementary to the target sequence. The basic scheme can be altered to suit a variety of gene-editing needs (28). The guide RNA directs the Cas9 nuclease to the targeted DNA region, which must contain a protospacer adjacent motif (PAM) at the 3′ end. The binding of Cas9 nuclease to the target region induces DNA double-strand breaks (DSBs), which subsequently trigger endogenous mechanisms to repair the DSBs. DSBs can be repaired either by non-homologous end joining (NHEJ) or homology-directed repair (HDR). In actively dividing human cells, NHEJ is the prevailing DNA repair mechanism, remedying 75% of naturally occurring DSBs, while HDR is responsible for the remaining 25% (31). NHEJ is an error-prone process and causes random DNA insertions or deletions (indels), which could generate frameshift mutations (32). Thus, NHEJ is useful when the resultant edits are intended to abolish gene expression. For precise gene edits such as single-base substitutions, a donor repair template is needed to shift the DNA repair pathways from NHEJ to HDR (33) (Figure 2A). HDR enhances the precision of CRISPR, but the issue remains that unlike NHEJ which is active throughout the cell cycle, HDR is only active at the G_2_ and S phases. This decreases the efficiency of HDR; and the problem is exacerbated in post-mitotic cells, which are not actively dividing (34).

The discovery of DNA base editors in 2016 offered an HDR-independent solution to the problem (35). These base editors utilize Cas9-nickase or dCas9 conjugated with deaminase to induce single-base transitions from C to T or A to G (36, 37) (Figure 2B). Prime editing expands the types of base substitutions that can be created by the base editors, using its dual-functioning guide RNA to prime the *synthesis* of DNA edits by a reverse transcriptase (Figure 2C). Instructed by a template embedded in the guide RNA, the reverse transcriptase can create precise indels or any of the 12 possible point mutations (C → T, G → A, A → G, T → C, C → A, C → G, G → C, G → T, A → C, A → T, T → A, and T → G) without the need for DSBs or an HDR template (37).

Precise single-base editing would be an important, clinically relevant modality of gene editing, as ~50% of disease-causing mutations are single-nucleotide substitutions rather than small indels. The Cas9-deaminase base editors may find use in correcting those mutations and treating the associated disorders (37, 38). For instance, a base editor has been shown to correct a mutation that caused Niemann-Pick disease type C and accumulation of lipids in mouse brain tissue (39). Prime editing is expected to surpass the base editors in therapeutic utility, as it could edit up to 89% of known genetic variants associated with human diseases (37).

## Generation of Cell and Animal models of HNDS Using CRISPR

Genes hold the blueprint for how the brain matures and functions. However, the roles of many genes in the developing human brain are still poorly understood, making the search for new HND treatments a difficult undertaking (40). *In vitro* and *in vivo* disease models are useful for understanding the molecular mechanisms and the pathogeneses of HNDs and exploring novel therapies. However, the generation of disease models using the conventional transgenesis technique, which introduces an altered version of a gene (harbored in a vector) into a host organism, is time-consuming and inefficient. Cells naturally reject foreign substances, so the expression of the mutant gene is usually lost after several rounds of cell division (41). CRISPR obviates this limitation, creating inheritable and permanent changes in nuclear (native) DNA (42).

CRISPR has been used to rapidly create *in vivo* and *in vitro* models to elucidate the pathogenetic mechanisms of genetic diseases and to identify potential treatments (Table 2). For instance, to facilitate the study on how the loss of *UBE3A*, which regulates synaptic development, in neurons leads to Angelman syndrome, *in vitro* and *in vivo* (rat) models were generated by knocking out *UBE3A* using CRISPR. *UBE3A*-deficient rats showed signs similar to what have been observed in patients with Angelman syndrome, namely cognitive and motor impairment. Neurons lacking functional *UBE3A* lose the ability to fire mature action potentials—the electrical signals that connect neurons and underpin the workings of the brain (43, 44). By silencing *UBE3A*, CRISPR has helped to pinpoint the genetic switch of neural circuits and the causal gene for Angelman syndrome.

**Table 2:** HNDs models generated by CRISPR.

**HNDs**	**Targeted gene**	**Species**	***In vitro/*** ***in vivo***	**Genetic alteration**	**Delivery method**	**Editing method**	**References**
Angelman syndrome	*UBE3A*	Human	*In vitro*	NHEJ-mediated gene knockout	Transfection	Cas9 and sgRNA	(43)
		Rat	*In vivo*	NHEJ-mediated gene knockout	Embryo microinjection	Cas9 and sgRNA	(44)
Lissencephaly	*Dcx*	Ferret	*In vivo*	NHEJ-mediated gene knockout	Embryo microinjection	Cas9 mRNA and sgRNA	(45)
	*Cdk5*	Ferret	*In vivo*	NHEJ-mediated gene knockout	*In utero* electroporation	Plasmid expressing Cas9 and sgRNA	(46)
Infantile neuronal ceroid lipofuscinoses	*PPT1*	Ovine	*In vivo*	HDR-mediated mutation	Zygote microinjection	Cas9 mRNA, sgRNA and HDR template (90 mer single-stranded oligodeoxynucleotide)	(47)

Mice and rats are popular choices of model organisms for studies of human diseases. However, they are not always compatible with human HNDs. Though the brains of humans, mice, and rats share the same general layout, they differ in some key aspects, making it impossible to create valid mouse or rat models for certain HNDs. The cortex of the human brain is heavily folded to house dense networks of neurons that perform high-level cognitive functions—an anatomical feature that is absent from the brains of mice and rats (48, 49). Beneath the cortical sheath, neurons are grouped by the genes they actively express into a variety of functional classes. Many of the neuron classes are conserved between humans and mice, but some putative counterparts were found to have notably varied patterns of gene expression (50). This means the molecular workings of some human brain diseases are species-specific and may only be accurately replicated in animals that are closely related to humans.

Medium-sized animals, such as sheep, monkeys, pigs, and ferrets resemble humans more closely than mice or rats and are therefore better model organisms for use in CRISPR-based studies of HNDs (51). For instance, CRISPR-mediated genome editing was applied to develop a ferret model to study lissencephaly, which causes loss of cortical folding in the human brain (45). In this study, a CRISPR system targeting *Dcx* was injected into single-cell ferret embryos, which were then implanted into surrogate females. This abolished the function of *Dcx* and resulted in the birth of ferrets who had smooth brains, confirming the importance of *Dcx* in enabling neuronal migration during cortical folding (45). Infants born with lissencephaly have small brains and severe intellectual disability (40). In another study, by delivering a CRISPR system using *in utero* electroporation, researchers proved that *Cdk5* can be another gene required for cortical folding, as knocking out this gene resulted in smooth-surfaced brains (46). Both studies used ferrets, as cortical folds are present in ferrets but not in rodents (45, 46).

It is evident that the choice of an animal model depends on the characteristics of the disease in question. For instance, a CRISPR-ovine model is the logical choice for infantile neuronal ceroid lipofuscinoses, where deleterious mutations in the palmitoyl-protein thioesterase 1 (*PPT1*) gene cause progressive death of nerve cells. The incurable disease affects children and severely reduces their life expectancy to ~10% of the average lifespan of humans, as death typically occurs at ~9 years of age. Sheep are more effective disease models than ferrets in this case, though both have brain structures similar to humans. The longer lifespans of sheep would allow us to thoroughly map out the development of the disease (47, 52, 53).

Overall, the studies curated in Table 1 have clearly shown the utility of CRISPR, when coupled with medium-sized animal models, in helping us to understand the pathogeneses of HNDs. However, the high costs, long breeding periods, ethical concerns, and strict regulations may still limit the use of those animal models in the future (51, 54).

## CRISPR-Mediated Treatment of Hereditary or Developmental Neurological Disorders

**Figure 3:**
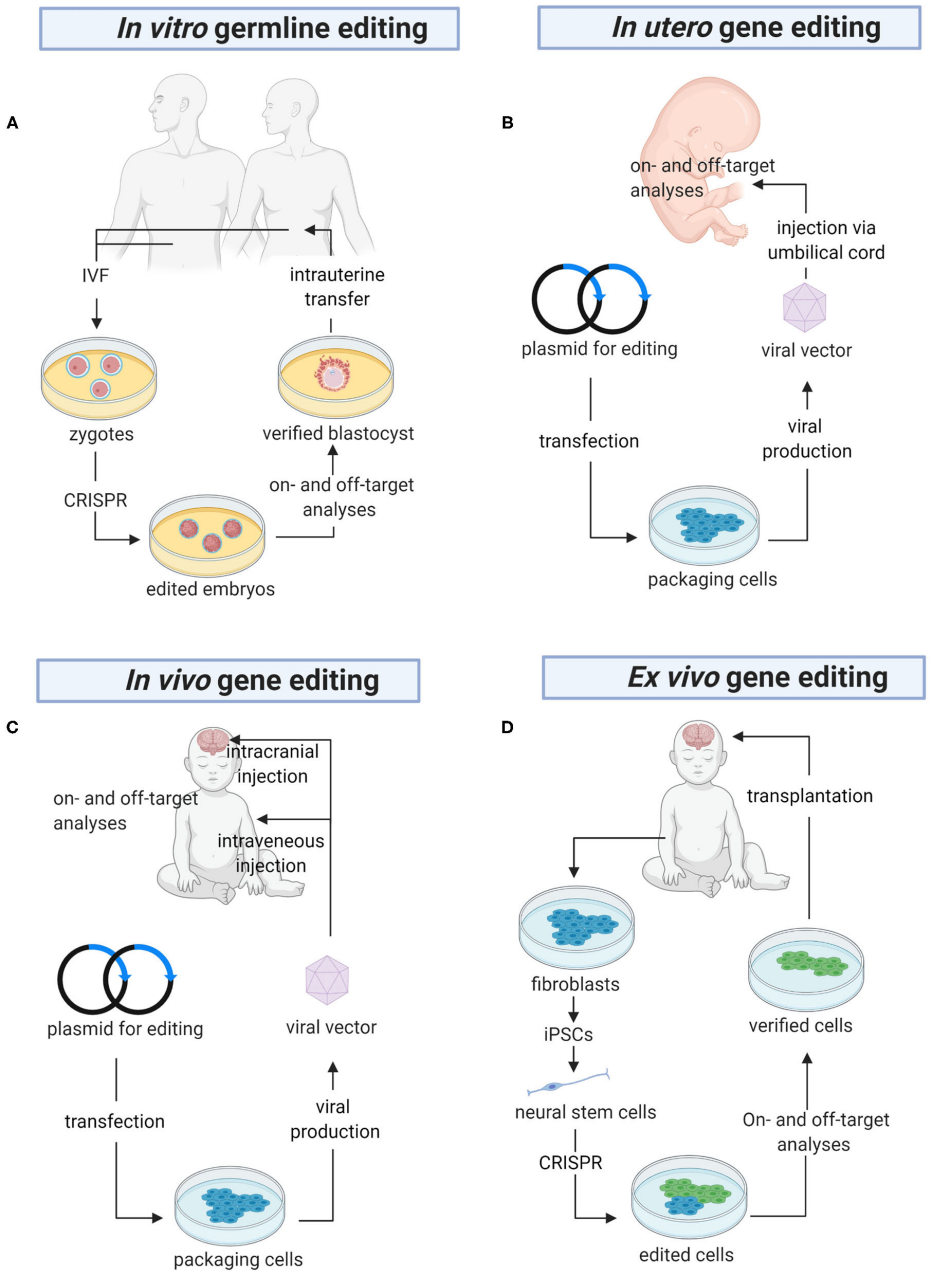
The future of gene editing in HNDs. **(A)** *In vitro* germline editing is initiated with the creation of zygotes. CRISPR constructs are microinjected into the zygotes, which are allowed to grow into embryos harboring the desired DNA edits. PGD is carried out to ensure there are no off-target mutations before the embryos are transferred into the uterus. **(B)** A viral vector harboring a genome editor is injected into the umbilical cord for direct delivery into the fetus. Alternatively, the editor can be delivered using a non-viral vector (not shown in the figure). Before the baby is born, a variety of tests will be performed to confirm on-target gene edits and detect off-target mutations. **(C)** CRISPR is packaged in a viral or non-viral vector for systemic delivery or direct injection into the brain. **(D)** *Ex vivo* gene editing begins with isolation of fibroblasts from the patients. The cells are reprogrammed into iPSCs, differentiated into neural stem cells, and CRISPR-edited. Then, the edited cells are analyzed for on- and off-target gene edits before they are transplanted into the brain. PGD, pre-implantation genetic diagnosis; IVF, *in vitro* fertilization; iPSCs, induced pluripotent stem cells (Created with BioRender.com).

Besides its potential in the generation of effective models of human diseases, CRISPR can also be used to treat HNDs (Table 3). Here we discuss in detail the pre-clinical findings reported by studies of a variety of HNDs, namely fragile X syndrome, Down syndrome, and sphingolipidoses. CRISPR-based therapy could be achieved using different approaches in the clinical settings, namely *in vitro* germline editing, *in utero* gene editing, and *in vivo* and *ex vivo* gene editing (Figure 3). We detail the rationales and challenges of different strategies for CRISPR editing of HND-causing DNA variants in Table 4.

**Table 3:** CRISPR-mediated treatment of HNDs.

**HNDs**	**Targeted gene**	**Species**	***In vitro/*** ***in vivo***	**Genetic alteration**	**Delivery method**	**Editing method**	**References**
Fragile X syndrome	*mGluR5*	Mouse	*In vivo*	NHEJ-mediated gene knockout	Intracranial injection	CRISPR–Gold Cas9 sgRNA RNPs	(12)
Down syndrome	Chromosome 21	Mouse, human	*In vitro*	CRISPR-mediated chromosome deletion	Transfection	Plasmid expressing Cas9 and sgRNA	(55)
Tay-Sachs	*HEXA*	Mouse	*In vivo*	cDNA-mediated Hex enzyme expression	Hydrodynamic injection	AAV-SaCas9 and AAV-*HEXM*-gRNA plasmids	(56)
	*HEXA* 1278 + TATC	Human	*In vitro*	TATC deletion	Transfection	Prime editing (PE3/PE3b plasmid, pegRNA plasmid, sgRNA plasmid)	(37)
Sandhoff disease	*HEXB*	Mouse	*In vivo*	cDNA-mediated Hex enzyme expression	Hydrodynamic injection	AAV-SaCas9 and AAV-*HEXM*-gRNAplasmids	(56)
Niemann-Pick disease type C	*NPC1* c.3182T>C	Mouse	*In vivo*	C → T	Retro-orbital injection	AAV-mediated cytosine base editor	(39)

**Table 4:** Advantages and disadvantages of different strategies of CRISPR-based gene editing in HNDs.

	**Advantages/rationales**	**Disadvantages/challenges**
**(A)** *In vitro* germline editing (57)	• The risk of affecting the mother is low. • Gene edits are not inheritable. • May allow parents who are homozygous carriers of recessively transmitted diseases to have a healthy child.	• Ethical, legal and social issues. • Its use is not justified as inherited genetic disorder can be prevented by embryo screening in most of the cases.
**(B)** *In utero* gene editing (58)	• Avoids the manifestation of life-threatening genetic diseases. • BBB is more permissive; vectors can be easily delivered to brain cells via systemic delivery. • Actively proliferating cells increase the efficiency of HDR. • Immune system can tolerate the gene editing system. • Decreases therapeutic dosing as the fetus is small in size.	• The safety of both the mother and the fetus should be ensured. • Off-target effects need careful evaluation in pre-clinical settings. • Difficult to determine the timing of intervention. • Risk of unintended germline editing (59).
**(C)** *In vivo* gene editing (post-natal)	• Ameliorates disease symptoms for conditions diagnosed after birth. • Poor engraftment of edited cells can be avoided. • Mother is not affected by the gene editing system.	• Presence of pre-existing immune response to the viral vector or CRISPR constructs, limiting the efficacy of repeat doses that may be necessary (58). • Important to select an appropriate vector to cross the BBB and target neuronal cells.
**(D)** *Ex vivo* gene editing (post-natal)	• Ameliorates disease symptoms for after birth diagnosis. • Precise selection of genetically modified cells without off-target mutations (60). • Minimal immune response. • Mother is not affected by the gene editing system.	• Time-consuming as the procedure is complicated. • Poor engraftment of edited cells (61).

### Fragile X Syndrome

**Figure 4:**
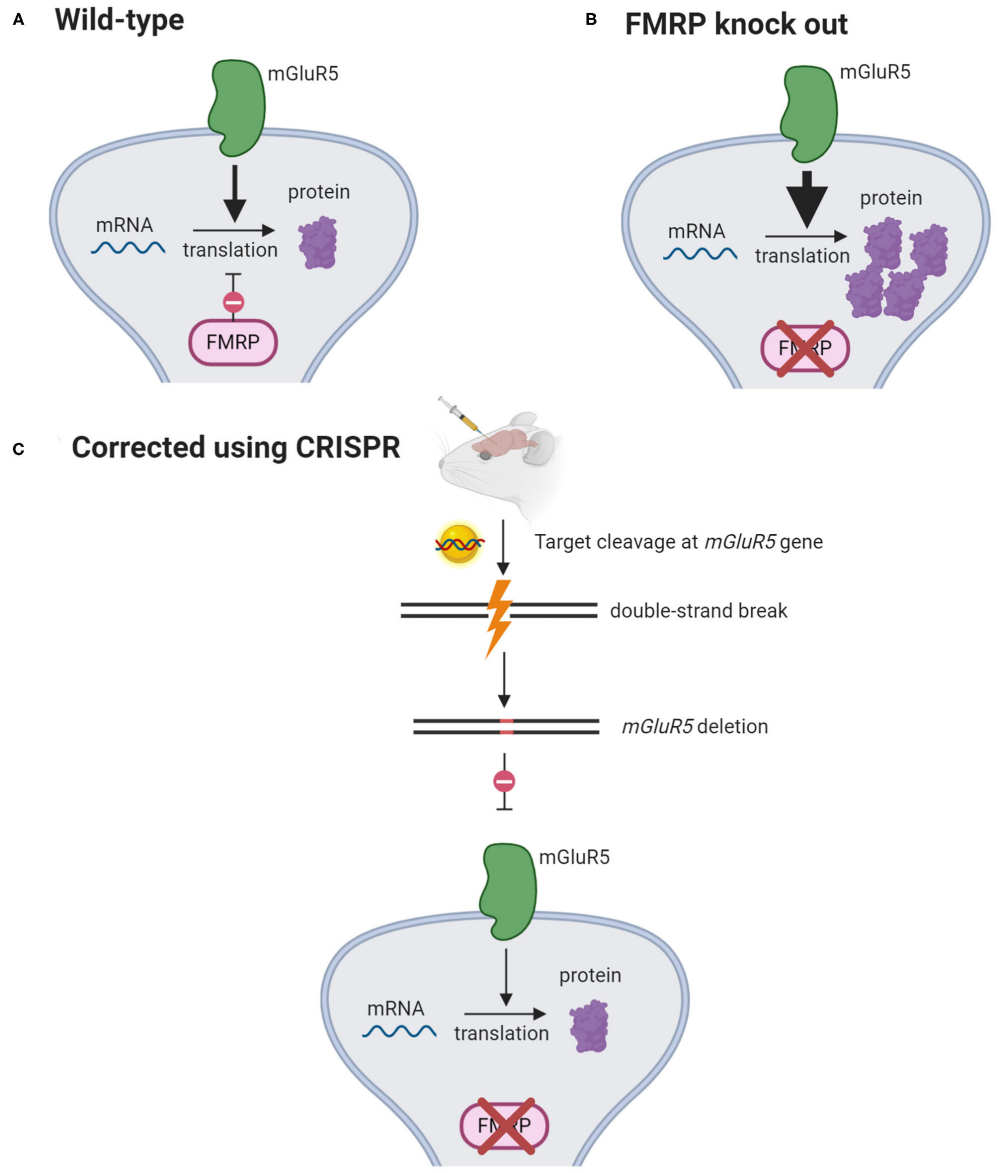
mGluR5 signaling reduction rescues fragile X syndrome in mice. **(A)** mGluR5 signaling activates protein synthesis. FMRP opposes mGluR5 and suppresses the translation of mRNAs into proteins. **(B)** In FMRP-knockout mice, mGluR5 signaling is exaggerated, causing excessive synthesis of proteins. **(C)** CRISPR-Gold is injected intracranially to knock out *mGlur5* gene. This has been shown to decrease mGlurR5 mRNAs by 40–50% and restore protein synthesis to its normal levels (12). (Created with BioRender.com).

CRISPR-Gold, a non-viral delivery system, was found to effectively edit *mGluR5*, an autism-associated gene, in a mouse model of fragile X syndrome. *mGluR5* editing reduces the signaling between brain cells and thus decreases the repetitive behaviors caused by this disorder (Figure 4). In the study that examined the therapeutic potential of CRISPR-Gold, the CRISPR system was injected directly into mouse brains to limit gene editing to the striatum, which mediates repetitive behaviors. As a result, *mGluR5* mRNA and protein levels were reduced by 40–50%, and this was sufficient to rescue the treated mice from repetitive behaviors (12). However, it could be a challenge for researchers to determine the extent of *mGluR5* reduction that would cause a similar effect in humans. Fragile X syndrome is associated with an imbalance of glutamatergic and GABAergic signaling (64). *mGluR5* serves a role in excitatory glutamatergic neurotransmission and completely knocking out this gene can further disrupt GABAergic signaling and impair human brain function (65, 66). Hence, the nuanced balance between the glutamatergic and GABAergic signals in the brain dictates the level of *mGluR5* inhibition that would be therapeutic in humans (67).

### Down Syndrome

Down syndrome, also known as trisomy 21, is caused by an error in cell division that leads to an extra chromosome 21 (68). It is a well-known genetic disorder that impairs neurodevelopment in newborns. The extra chromosome 21 causes overexpression of >100 genes that drive brain development or function (69). Several gene editing strategies, including CRISPR, have been applied to eliminate the surplus chromosome (70). For instance, two gRNAs were designed to target repetitive sequences at the long arm of chromosome 21, induce cleavage at multiple sites, and eliminate the whole chromosome. The deletion of an entire chromosome is challenging as it is difficult to efficiently induce multiple DNA cleavages. Although the initial trial of the chromosome-removing strategy was successful in stem cells derived from patients with Down syndrome, the same outcome was not replicable in embryos, probably because chromosomal deletion was lethal to embryonic cells (55). Recently, two alternative strategies were proposed. The suggestions were aimed at inactivating instead of deleting the extra chromosome 21 (71). Guided by sgRNAs, the Cas9 nuclease could home in on and cut off the Down syndrome critical regions in chromosome 21, which harbor the culprit genes that cause Down syndrome and inhibit neuronal development. Alternatively, the enzyme could edit out a non-functional segment within chromosome 21 in exchange for a regulatory DNA construct which contains *XIST* that inactivates the chromosome (71, 72). With this proposed approach, chromosomal inactivation by *XIST* which normally occurs at the pluripotent stage could be induced in non-pluripotent neural stem cells and differentiated neurons (73). Both the proposed methods could rescue neurogenesis and improve cognitive performance in Down syndrome patients.

### Sphingolipidoses

CRISPR has also demonstrated its genome editing efficacy in mouse models of Tay-Sachs and Sandhoff diseases. Mutations in *HEXA*, which encodes the Hex α subunit, lead to Tay-Sachs disease while mutations in *HEXB*, which encodes the Hex β subunit, cause Sandhoff disease. Mutations in the *HEXA* and *HEXB* genes reduce the activity of beta-hexosaminidase, which breaks down G_M2_ ganglioside, a normal component of the neuronal membrane. As a result, G_M2_ ganglioside accumulates to a level which is toxic to neurons in the brain and the spinal cord, causing intellectual disability and seizures (74). Instead of targeting the brain, a CRISPR system delivered by AAV was used to turn hepatocytes into machinery that produces a modified human Hex μ subunit, by integrating cDNA encoding the protein into the albumin gene. The enzymes expressed and secreted from the edited hepatocytes were then carried by the bloodstream to the brain to break down G_M2_ ganglioside (56).

With new techniques being rapidly developed, several alternative strategies—one of which being prime editing—have become available for editing *HEXA* mutations in Tay-Sachs disease (37). The most common mutation found in patients with Tay-Sachs disease is a 4-bp insertion, i.e., TATC in exon 11 of the *HEXA* gene (75). In an *in vitro* model, prime editing was shown to correct the mutation by removing the 4-bp insertion without DSB (37). To determine which CRISPR system is optimal, criteria such as safety, costs, delivery vectors, and how well the system works in cells should be considered. Most importantly, more supporting evidence should be garnered from pre-clinical studies before moving into clinical trials.

## Gene-Editing to Study Drug Responsiveness

Caffeine, an antagonist of adenosine A1 (ADORA1) and A2A receptors (ADORA2A), is a key modality of the management of apnoea of prematurity. Administration of caffeine in pre-mature, apneic infants was found to improve symptoms and significantly reduce death rates and the severity of neurocognitive impairment (76). Caffeine has also been shown to have a variety of neuroprotective effects *in vitro* and *in vivo*. It protects against cell death and preserves background electrical activity in the hypoxic-ischemic brains (77–80) and enhances the connection between neurons by activating genes that control neuron projection. This is especially important to the developing brains of infants. Together, the protective mechanisms of caffeine act to improve neurodevelopment in preterm infants (81).

Individual genetic differences can affect the pharmacology of some drugs and cause inter-individual variability in drug response. This would affect drug therapy outcomes. Because of genetic variation, not all infants given caffeine will respond optimally to the drug. Rs16851030, a DNA variant located in the 3′-untranslated region of the *ADORA1* gene, was shown to adversely affect the outcome of caffeine therapy. A prospective case-control study was conducted to assess the variability of caffeine sensitivity in relation to single-nucleotide variants in the *ADORA1* gene. All infants who were >28 weeks old and homozygous for the rs16851030 reference C-allele were found to have responded favorably to caffeine therapy, in comparison with a 57% response rate among those harboring the alternate T-allele. The discrepancies in the treatment outcomes may be due to the influence of rs16851030 on the expression of the adenosine A1 receptor (16); but this remains an unconfirmed theory. Furthermore, we do not know whether the genetic variation in caffeine response could be overcome by dosage adjustment (82). In caffeine-sensitive individuals, the augmented effects of caffeine could be offset by lowering caffeine doses to avoid toxicity, such as tachycardia and seizure (83). Conversely, individuals who are less caffeine-responsive may benefit from higher caffeine doses to reduce the risk of apnea and to prevent complications such as hypoxia-induced brain damage. CRISPR has been used to create a mutant breast cancer cell line with a single base edit to elucidate the mechanism of drug resistance (14). Therefore, by creating a cell-based model using CRISPR and treating the cells carrying the C- or T-allele with different concentrations of caffeine, we could then gauge whether the underlying genetic influence could be overcome by adjustments to the dosage of caffeine. The resultant findings would be valuable for optimizing the management of apnea of prematurity and improving neurodevelopmental outcomes in preterm infants.

Using a base editor to investigate how rs16851030 affects the outcome of caffeine therapy could be challenging as there are multiple Cs around the target C (in brackets) within the editing window, as shown in the flanking sequences of rs16851030, TCTTAGATGTTGGTGGTGCAGC[C/T]CCAGGACCAAGCTTA**AGG**AGAG. The editing of additional Cs can cause harmful effects. CRISPR base editors with narrow editing windows were reported recently but they still would not be able to precisely edit the target C, owing to bystander effects (84, 85); and the editing may result in a non-T (86, 87). Prime editing would be a fitting alternative to the base editors (Figure 2C), as it creates precise point mutations by directly copying the desired gene edits into the target DNA segments (37). Another advantage of prime editing is that unlike the other base editors, it can perform gene editing in post-mitotic cells, including neurons in the brain (37).

## Limitations of CRISPR and the Way Forward

In this section, we detail the roadblocks to CRISPR attaining its maximal utility in neuroscience research: off-target effects, potential difficulties in crossing the blood-brain barrier, and immunogenicity of Cas9 and vulnerability of neuronal cells to the adverse effect of CRISPR editing (or the system that delivers it).

### Off-Target Effects

The specificity of CRISPR is ensured by its companion gRNA, which consists of sequences complementary to the target DNA region. However, the specificity is not absolute, and unintended binding between gRNA and non-target DNA sequences is possible. Off-target activity must be avoided because it can lead to adverse side effects. Current tactics for curbing off-target editing have focused on two key aspects of how CRISPR operates, i.e., the need for specific gRNAs, and the fundamental gene editing mechanics.

DISCOVER-Seq (discovery of *in situ* Cas off-targets and verification by sequencing) is a tool for detecting possible *in vitro* or *in vivo* off-targets of CRISPR, helping researchers to validate the guide RNAs they have designed *in silico*. By checking the interaction between a DSB repair protein, MRE11, with Cas9 cut sites, DISCOVER-Seq can identify the exact locations in the genome where a cut has been made by CRISPR. The MRE11-bound DNA segments are captured by chromatin immunoprecipitation and sequenced on a high-throughput platform. DISCOVER-Seq is superior to other tools as it can be used to detect off-target events *in vivo* (88). This may in then inform corresponding strategies to eradicate the off-target editing.

Prime editing overhauls the mechanism of base editing and could be an option that is relatively free of off-target editing. It was reported to have increased target specificity (37). However, the safety and efficacy of prime editors in neuronal cells are still unclear. Further studies are needed to explore the utility of this newly developed gene editor in the neuroscience space.

### Crossing the Blood-Brain Barrier

For gene expression studies and treatment of HNDs, the main challenge is to deliver a CRISPR system across the BBB. The BBB prevents the entry of foreign substances into the brain, including toxins and pathogens (89). The protective mechanism is a two-edged sword, as it also cuts off the access of CRISPR systems to the brain. There are several strategies to tackle the BBB, such as viral delivery and nanoparticles. AAV is a popular method for shipping CRISPR expression constructs to the target brain cells. To make room for CRISPR, the virus is emptied of its protein-coding genes, leaving only the capsids and the sequences that regulate DNA replication. The passage of AAV-CRISPR across the BBB is made possible by the inherent ability of viruses to bind to and invade host cells (90).

However, wild-type AAVs are inefficient in crossing the BBB and need direct injection into the brain (91). Besides, they have low transduction efficiency *in vitro* and *in vivo* (92, 93). To counter the drawbacks, a 7-mer peptide, PHP.B, was inserted into the capsids of wild-type AAVs to facilitate the penetration of BBB and to increase transduction efficiency in neuronal cells (93, 94). However, a high viral load of AAV-PHP.B would be required (≥1 ×10^12^ vg per adult mouse) for genetic modification in the brain, and this translates into a high risk of immune reactions. With the development of AAV-PHP.eB, which varies from AAV-PHP.B at only two amino acids adjacent to the initial 7-mer peptide insertion, neuronal cells can be edited using a lower viral load (95). Some studies showed that PHP.B and PHP.eB require the LY6A receptor (lymphocyte antigen 6 complex) to reach the mouse brain. *Ly6a* disruption decreases, while *Ly6a* overexpression enhances, transduction efficiency (96, 97). Nonetheless, this mechanism utilized by AAVs to cross the BBB is mouse-specific and there is no direct homolog to *Ly6a* in humans. Further experiments should focus on pinpointing a gene which can be targeted to increase AAV transduction in the human brain (98).

Another way to deliver the CRISPR system across the BBB has been developed recently using *in vitro* BBB models and holds promises in the eradication of neuroHIV. It was achieved by packaging the CRISPR system bound with magneto-electric nanoparticles (MENPs) in a nanoformulation. A magnetic field was then applied on the nanoformulation to release CRISPR from the surfaces of MENPs and to facilitate cell uptake. This would then result in intracellular release of CRISPR and inhibition of HIV (99). Neonates acquire neuroHIV when the virus enters their brains, and this could delay their brain development (100, 101). Although HIV is not an inherited disease, the same approach to delivery across the BBB could be applied in the treatment of HNDs. However, it is unclear whether the BBB has fully formed during the neonatal period (89). This would affect the concentration of a CRISPR system to be safely delivered into the brain. Future efforts should focus on determining the optimum concentration of CRISPR before this technique can be adopted clinically.

### Immunogenicity of Cas9 and Vulnerability of Neuronal Cells to the Adverse Effect of CRISPR Delivery Systems

The most common Cas9 orthologs are derived from *Staphylococcus aureus* (SaCas9) and *Streptococcus pyogenes* (SpCas9) (102). Because of their bacterial origins, Cas9 proteins face pre-existing adaptive immune responses in humans. Antibodies against SaCas9 and SpCas9 have been detected in 86 and 73%, respectively, of the serum samples obtained from cord blood donors (103). The potential immunogenicity of Cas9 proteins warrants caution in future clinical trials examining the use of CRISPR in neonates.

Besides, the delivery methods of CRISPR systems may also induce immune responses and impact neuronal cells. Viral vectors are commonly used to deliver CRISPR constructs across the BBB (12, 104). However, viral delivery causes protracted CRISPR expression, which is toxic to neuronal cells and alters neuronal phenotypes (12). Moreover, it has been demonstrated that persistent Cas9 expression elicits cytotoxic immune response, which removes genetically edited cells. The removal of modified cells in the brain could lead to adverse consequences, as brain cells have limited capacity to regenerate (105). The finding also means that CRISPR edits are not necessarily permanent, so repeat administration of CRISPR therapy would be required (106). Hence, non-viral delivery methods have received great interest recently; for instance, gold nanoparticles have been used to deliver Cas9 ribonucleoproteins targeting *mGluR5* in a mouse model of fragile X syndrome (12). Gold nanoparticles are safe, as they were not found to cause cytotoxicity or changes to neuronal functions at low doses. Also, they did not induce immune responses—a common problem arising from the delivery of CRISPR systems via viral methods (12).

Overall, nanoparticles seem an ideal carrier for delivering CRISPR systems into brain cells. Nanoparticles are versatile as their surfaces can be engineered to target specific cells. For instance, gold nanoparticles coated with exosomes have been shown to be able to cross the BBB via endocytosis (107, 108). To selectively target brain cells, the exosomes can be modified with a neuron-specific peptide derived from the rabies virus glycoprotein. This peptide specifically binds to the acetylcholine receptors expressed by neuronal cells (109, 110). Therefore, by modifying the surfaces of nanoparticles, we can ensure a CRISPR system is able to pass through the BBB and reach its target brain cells.

## Ethical and Future Perspectives

In the last few years, CRISPR-driven research is rapidly increasing, and new cell and animal models have been created to elucidate the pathogenesis of HNDs. This is important groundwork for future research into new therapies. The family of CRISPR-Cas9 gene editors has been growing steadily. A variety of base editors and prime editors are continually being discovered that may improve the precision and efficacy of gene editing. Studies trialing the gene editors have resulted in various success rates. The simple mechanics of CRISPR make it a robust gene-editing tool; however, off-target editing is still a major concern and could have severe consequences (111). The safety of CRISPR editing should be guaranteed in two aspects. First, enhancing the precision of gene editing should remain at the core of future CRISPR-centric research. It may be helpful to pinpoint “hotspots” in the genome where off-target edits are most likely to arise. This may then lead to strategies that can effectively curb off-target editing in those DNA segments. Second, the chosen mode of CRISPR delivery should be non-toxic to neuronal cells and non-immunogenic. The body's immune response may suppress CRISPR gene therapy, and pose a health risk to the person receiving the treatment. Screening for potential immunological or allergic reactions to CRISPR should be performed before commencing therapy.

An appealing use of the CRISPR technique would be pre-emptive *in utero* editing of pathogenic gene mutations coupled with prenatal genetic testing (112–114). This would be better than delaying gene therapy until after birth, when the disease would have become manifest and the damage established. Some of the HNDs develop before birth; for instance, lissencephaly impairs cortical folding and is irreversible once the prenatal brain development is completed (115).

However, a long road lies ahead for the adoption of CRISPR-based gene editing in the clinics. What is therapeutic and what is not; or defining which genetic diseases should take priority for CRISPR therapy, are some difficult choices to make even in settings with relatively abundant health care resources. CRISPR therapy is likely to be costly—some estimates have priced it at USD $0.5 to $2 million, so funding it would be a challenge for most countries or insurance companies. Owing to the exorbitant costs of emerging gene therapies, health insurers may become increasingly selective in choosing their clients, excluding those diagnosed with “pre-existing conditions” (116, 117).

Ethical concerns are also important considerations before CRISPR can be used in humans. Genome editing in clinical settings is currently limited to somatic cells, as this is less likely than germline editing to be misused for non-ethical purposes. Potential problems may arise if “designer babies” are created using CRISPR. For instance, undetected off-target effects can be passed down to future generations and the undesirable negative consequences may be grave. Other ethical considerations include using CRISPR to achieve better phenotypic characteristics, such as height, intelligence, and athletic performance. This highlights the need for strict regulations and judicial frameworks on human germline editing. The National Institute of Health supports the call for an international moratorium on human germline editing in the clinical settings until certain conditions are met (118). Global discussions involving scientists and ethicists are needed to address how germline editing should be performed and ethically acceptable before the moratorium can be lifted.

In summary, CRISPR is an effective research tool for studying HNDs. If important safety and ethical concerns can be addressed, it has immense potential as a new treatment modality for HNDs. We expect more established CRISPR-based treatment strategies that bring new hopes for HNDs in the future.

## Author Contributions

All authors listed have made a substantial, direct and intellectual contribution to the work, and approved it for publication.

## Conflict of Interest

The authors declare that the research was conducted in the absence of any commercial or financial relationships that could be construed as a potential conflict of interest.
